# Editorial: Policies, programs and treatments on smoking cessation during the COVID-19 pandemic

**DOI:** 10.3389/fpubh.2024.1433524

**Published:** 2024-07-08

**Authors:** David C. N. Wong, Yim Wah Mak, Yong Zhao, Vaughan W. Rees

**Affiliations:** ^1^Research Office, Tung Wah College, Kowloon, Hong Kong SAR, China; ^2^School of Nursing, Hong Kong Polytechnic University, Kowloon, Hong Kong SAR, China; ^3^School of Public Health and Management, Chongqing Medical University, Chongqing, China; ^4^Harvard T. H. Chan School of Public Health, Boston, MA, United States

**Keywords:** COVID-19, tobacco and tobacco product, e-cigarette (e-cig), smoking cessation, perception, policy, treatment, smoking

The global tobacco pandemic is a key driver of the world's leading causes of morbidity and mortality results in more than 8 million deaths yearly, including active and passive smokers (1). Over the past few decades, many countries have implemented interventions and services that prevent tobacco use and help people stop smoking. However, certain population groups in different background still face challenges in attempting to quit successfully.

The COVID-19 pandemic imposed disruption across health services and public health interventions, while changing the working and domestic routines of people globally. The World Health Organization (WHO) recognized that the outbreak had constituted a public health emergency of international concern from 30 January, 2020 to 4 May 2023 (2, 3). Given the rapid spread of the disease, and in response to the WHO's declaration of a public health emergency, many countries imposed stringent social distancing measures over the period, including travel restrictions, lockdowns and quarantines. Many governments introduced mandatory mask-wearing requirements in public areas. Infection with COVID-19 posed special risks for people with a history of smoking, with studies pointing to greater disease severity, likelihood of hospitalization, and death, relative to people who do not smoke (4). Yet the disruptions caused by COVID-19 were associated with increased smoking behavior, prompted by feelings of isolation, stress and depression (5), as well as the absence of smokefree laws that reduce smoking and second-hand smoke exposure in the workplace and other public settings, but not in private homes where many people were confined (6). Unfortunately, the disruptive effect of COVID-19 on increased smoking behavior also impacted the availability of traditional smoking cessation support services, creating even greater challenges for people who smoke and those who work to protect them.

This Research Topic brings together a collection of five manuscripts authored by leading public health scientists with expertise on. These manuscripts cover a diverse range of topics including perceptions and intentions regarding the use of alternative tobacco products (including e-cigarettes and heated tobacco products), the association between smoking and COVID-19 reinfection, and the impact of mask-wearing policy on smoking behaviors across different geographic locations.

Two papers examine the association between tobacco use and COVID-19 infection or re-infection. A multi-country online survey conducted by Sabbagh et al., found a positive association between COVID-19 infection and the use of e-cigarettes among adolescents and young adults. Using electronic health records (U.S. HealthJump database and the COVID-19 Research Database Consortium), Ando et al. found an association between COVID-19 reinfection and smoking among patients with COVID-19, affecting the rate of symptomatic reinfections.

Three studies address perceptions and factors associated with tobacco use in different population groups during the COVID-19 pandemic. Sabbagh et al. conducted a multi-country online survey on adolescents and young adults and found that ~30% of current smokers, who use either regular cigarettes or e-cigarettes, reported reduced tobacco consumption during the COVID-19 pandemic. Those who have lower levels of anxiety or a negative attitude toward smoking were less likely to smoke. Deng et al. conducted an online survey in Chongqing, China and reported that, during the COVID-19 pandemic, participants in general had a negative attitude toward e-cigarettes. The survey also found that medical professionals were less likely to use e-cigarettes. In a U.S. online survey, Sharma et al. found that *IQOS* heated tobacco products, held greater appeal than combusted cigarettes, especially for current smokers and those who perceive lower risks from COVID-19.

Furthermore, Sun et al. conducted an observational study and examined smoking behaviors of pedestrians at ten outdoor smoking hotspots in Hong Kong SAR, China for 33 months during the COVID-19 pandemic. They found that outdoor smoking rates decreased immediately after the implementation of mandatory mask-wearing regulation in public areas of the region, although it was not specifically designed as a tobacco control measure. However, it rebounded to pre-COVID-19 levels, probably due to the weak law enforcement that affected the compliance of people to abide by the COVID-19 regulations.

The papers in this Research Topic provide insights into the perceptions and smoking behaviors of different population groups during the COVID-19 pandemic. The scientific evidence presented establishes a positive association between smoking and COVID-19 infection/reinfection, while the presence of health threats, such as a pandemic, raises general health concerns in different population groups and promoted interest in quitting or in the use of reduced exposure tobacco products. These observations provide valuable insights for developing new strategies in campaigns aimed at preventing tobacco use and promoting smoking cessation in a complex global environment, subject to disruption from events such as infectious disease, climate change, political unrest, and mass migration.

## Author contributions

DW: Writing – original draft, Writing – review & editing. YM: Writing – review & editing. YZ: Writing – review & editing. VR: Writing – review & editing.
